# Determinants of Exclusive Breastfeeding for the First 6 Months Among Children Under Five in Internally Displaced Persons Camps in Eastern Democratic Republic of Congo; a Cross‐Sectional Study

**DOI:** 10.1002/puh2.70341

**Published:** 2026-08-11

**Authors:** Alimasi Kashafali, Abel Aizeque, Zubayer Shams, Kanza Farhan, Amidu Alhassan, Bilal Ahmad, Innocent Mufungizi, Frank Offei Odonkor, Elie Kihanduka, Christian Tague, Calvin R. Wei, Francois Rhugendabanga, Rodrigue Fikiri Bavurhe, Hugues Cakirwa, Fabien Balagizi, Aymar Akilimali, Freddy Zihindula, Michée Sanza Kanda, Amos Kipkorir Langat, Medical Research Circle (MedReC) Collaborators

**Affiliations:** ^1^ Department of Research Medical Research Circle (MedReC) Goma D. R. Congo; ^2^ Faculty of Medicine Official University of Bukavu Bukavu D. R. Congo; ^3^ Faculty of Health Sciences Catholic University of Mozambique Beira Mozambique; ^4^ Brunel Medical School Brunel University London UK; ^5^ Jinnah Sindh Medical University Karachi Pakistan; ^6^ Department of Adult Health College of Health and Allied Sciences University of Cape Coast Cape Coast Ghana; ^7^ Department of Medicine Shaikh Khalifa Bin Zayed Al‐Nahyan Medical and Dental College Lahore Pakistan; ^8^ Department of Research and Development Shing Huei Group Taipei Taiwan; ^9^ Faculty of Medicine University of Goma Goma D. R. Congo; ^10^ Faculty of Medicine Evangelical University in Africa Bukavu D. R. Congo; ^11^ School of Public Health Kalinga Institute of Industrial Technology Bhubaneswar India; ^12^ Pan African University Institute for Basic Sciences Technology and Innovation Nairobi Kenya

**Keywords:** children under five, cross‐sectional study, Democratic Republic of Congo, exclusive breastfeeding, internally displaced persons camps

## Abstract

**Introduction:**

Giving only breast milk in the first 6 months lowers the chances of childhood diseases and death. Still, it is difficult to promote ideal breastfeeding among internally displaced persons (IDPs) in camps. This research is intended to find out how many mothers are feeding their children under five with only breast milk in IDPs camps in Goma, eastern Democratic Republic of Congo (DRC).

**Methods:**

Between November 2024 and February 2025, an analytical cross‐sectional study was conducted in five major IDPs camps in Goma. A total of 407 eligible mothers or primary caregivers were recruited and participated in the study using a structured and pre‐tested questionnaire. Data were collected on sociodemographic characteristics, including maternal age, place of residence, and infant feeding practices. The chi‐square (*χ* ^2^) test was employed to assess associations between exclusive breastfeeding (EBF) and selected explanatory variables, with statistical significance determined at *p* < 0.05.

**Results:**

The prevalence of EBF was 20.9%. Significant associations were observed between EBF and maternal age (*χ* ^2^ = 18.8 and *p* < 0.05), educational level (*p* < 0.05), marital status (*p* < 0.05), presence of both parents (*p* < 0.05), prematurity status (*χ* ^2^ = 7.64 and *p* = 0.022), and source of household income (*χ* ^2^ = 28.3 and *p* < 0.001). Higher EBF rates were reported among younger mothers, those with higher education, married women, and households receiving humanitarian aid (51.6%) compared to those relying on trade (16.2%). No significant associations were found between EBF and child's sex (*χ* ^2^ = 0.177 and *p* = 0.674), camp of residence (*χ* ^2^ = 0.688 and *p* = 0.953), or household size (*χ* ^2^ = 3.78 and *p* = 0.052).

**Conclusion:**

IDPs camps demonstrate markedly low levels of exclusive breastfeeding, highlighting significant disparities between displaced populations and the general population. These findings underscore the need for targeted, context‐specific interventions, including strengthened maternal education, increased paternal support, and enhanced breastfeeding support programs, to improve infant health outcomes in humanitarian and crisis settings.

## Graphical abstract

Eastern Democratic Republic of Congo's IDPs camps in Goma have very few mothers who are exclusively breastfeeding their children, which is less than half of what is considered the standard by both national and international guidelines.

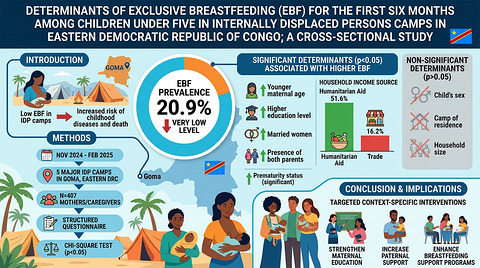

## 1 Introduction

Taking in only mother's milk in the first 6 months is considered vital for a healthy, well‐developed, and alive infant. According to the World Health Organization (WHO), babies should get only breast milk and not any other drinks, including water, up to 6 months unless it is absolutely required [1]. Protecting babies with exclusive breastfeeding (EBF) can decrease their risk of illness and death because it protects against illnesses such as diarrhea and pneumonia that are some of the leading reasons for death among kids within 5 years in poor parts of the world [2]. Although it brings great benefits, the world is still seeing less EBF than the recommendations. Many social, economic, cultural, and health problems are present in sub‐Saharan Africa, including the Democratic Republic of Congo (DRC), and these obstacles make breastfeeding difficult to practice [3, 4]. It becomes more difficult for women and children in internally displaced persons (IDPs) camps because they may experience additional problems from conflict, forced migration, little food, lack of medical help, and the emotional burden they face.

The eastern part of the country has been involved in continuous armed struggles for many years, and this has resulted in many people losing their homes [5]. Because most IDPs camps are packed and have few supplies, it is tough for newborns to be fed only mother's milk. Women living in detention centers might have problems due to their health, lack of privacy, difficulties learning how to breastfeed, and the spread of mistaken beliefs, making it harder for them to only breastfeed [6]. Even though studies on EBF mainly concern more permanent societies [7, 8], there is not much research on what exactly impacts this in displaced communities in the D. R. Congo. Knowing these points helps create public health interventions that suit a crisis. The study aims to discover the motives that influence mothers to breastfeed their children who are under five in IDPs camps in the eastern region of DRC for the first 6 months. Insights are aimed at steering measures that benefit and secure the safety and health of mothers and kids from vulnerable backgrounds.

## 2 Methodology

### 2.1 Study Design and Setting

**FIGURE 1:**
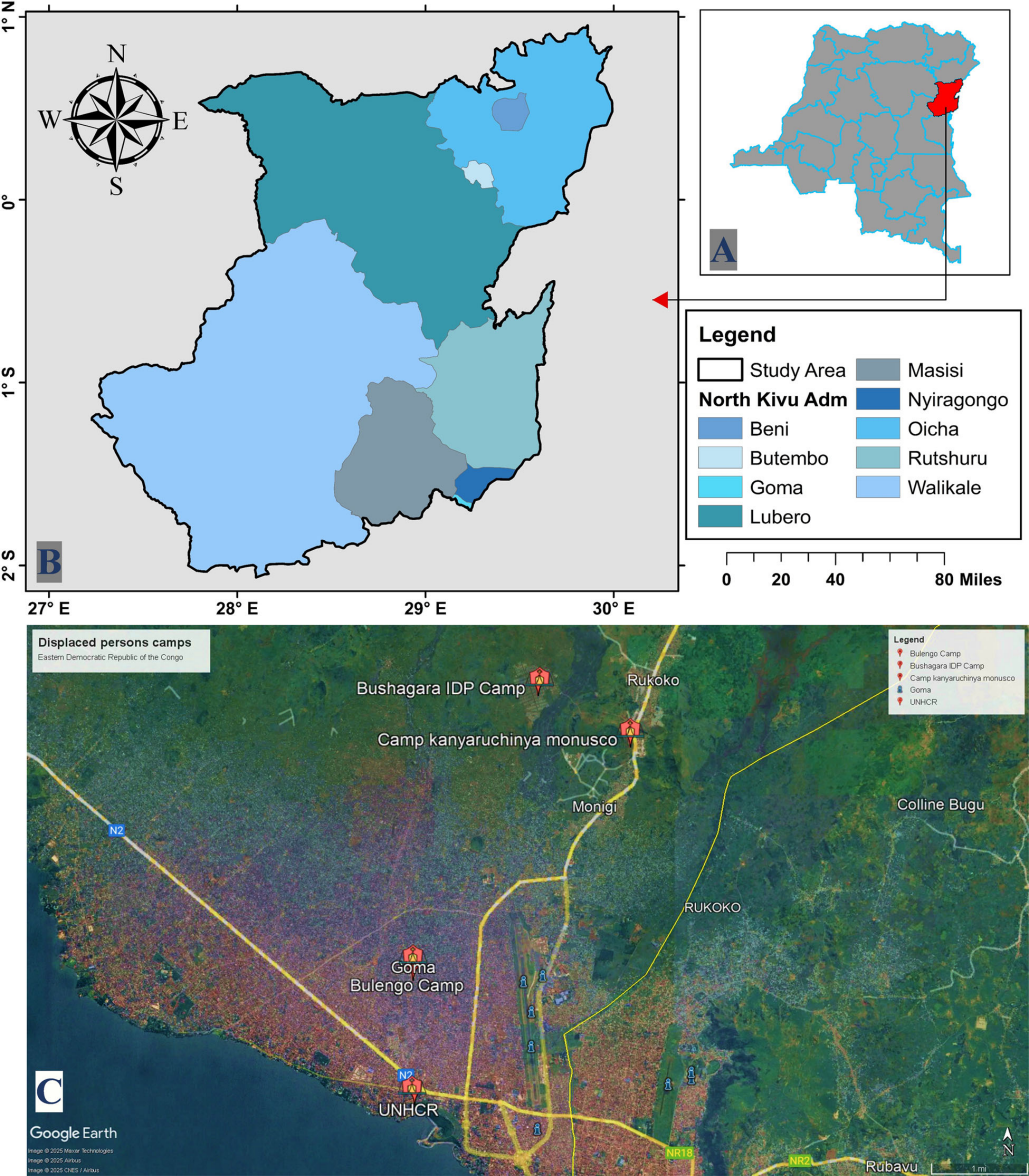
Location map of the study area: (A) Democratic Republic of Congo; (B) North Kivu Province; and (C) Satellite image showing the location of refugee camps.

This analytical cross‐sectional study was conducted over a period of 4 months, from November 1, 2024, to February 27, 2025, in five main IDPs camps (Rusayo, Bulengo, Don Bosco, Kanyaruchinya, and Lushagala) in the city of Goma, one of the big cities in the eastern region of the DRC (Figure 1). These camps are the main camps that host thousands of displaced populations due to the ongoing and persistent armed conflicts in the eastern region of the DRC, and these camps present precarious conditions in terms of food security, access to healthcare, and health infrastructure.

### 2.2 Study Populations

The population of this study consisted of mothers or guardians with children under 5 years old residing in the selected main camps.

### 2.3 Eligibility Criteria

Participants eligible for this study were those who had resided in the camp for at least 3 years, who had given birth within the last 5 years, and who agreed to participate in the study after receiving a clear explanation of the study objectives.

Exclusion criteria included participants who had lived in the camps for less than 3 years, participants with twins or triplets (to reduce bias related to specific breastfeeding practices), children with major congenital malformations or known chronic conditions affecting feeding, and children whose mother or guardian did not consent to participate in the study.

**Summary Box**

**How might this study affect research, practice, or policy?**

The study reveals that breastfeeding alone is very uncommon among people living in Goma, DRC, who are displaced after flight disruptions. It proves why it is important for humanitarian groups and those making decisions to handle emergencies to promote and support breastfeeding in their plans. Results from the research could help create programs for children that consider the unique roles of parents in society. This work also suggests that researchers should investigate how under‐breastfeeding in crisis situations affects people's long‐term health. Helping mothers increase their education and providing adequate support programs might be useful in improving the well‐being of infants and lowering their mortality rates.

**What this study adds?**

This work gives useful information about the rate of exclusive breastfeeding among people in camps for displaced persons in eastern DRC. The evidence points out that only one in five children in the region was exclusively breastfed, which means the goals are far from being achieved. In addition, it points out important influences such as mother's education, marriage, family backing, and sources of household income. This study provides important information about breastfeeding by describing real cases in a humanitarian setting that is not usually covered in the field. The study argues that immediate help should be given considering these mothers’ unique socioeconomic and environmental situations.

**What is already known on this topic?**

Giving only breast milk for the first 6 months increases a baby's chances of survival and reduces the chances of getting many childhood diseases. Various efforts are being carried out around the world to help increase the use of EBF, mainly in low‐ and middle‐income nations. People displaced by conflict or suffering from instability usually have major hurdles in breastfeeding properly. It was suggested by previous studies that EBF rates decrease when people experience stress, lack support, and cannot get enough health advice. Still, there are not many or complete reports on how internally displaced people in sub‐Saharan Africa, especially in the DRC, practice breastfeeding.

### 2.4 Sample Size Determination

The sample size was determined using Cochran's formula for single proportions, assuming a 95% confidence level, a prevalence of EBF of 37.5%, and a 5% margin of error. The minimum sample size obtained was 360. After adjusting for a 15% nonresponse rate, the final required sample size was 424 participants.

### 2.5 Collection of Data

Convenience sampling was the sampling technique used in this study. Data collection for this study was conducted using a structured questionnaire, administered face‐to‐face by interviewers trained by the research team and fluent in local languages (Kiswahili and French). This questionnaire was pre‐tested on a small number of people living in a camp that was not selected for this study to ensure clarity, cultural relevance, and content validity of the questionnaire. The questionnaire included: the respondent's sociodemographic characteristics (age, education level, marital status, employment, and parity), the child's characteristics (sex, age, and birth order), the mother's knowledge and attitudes toward EBF, breastfeeding practices during the first 6 months of the child's life, and environmental factors (access to health services, nutritional assistance, and family or community support).

### 2.6 Data Entry and Data Analysis

Data were entered and cleaned using Microsoft Excel and analyzed using IBM SPSS Statistics V25.0. Data were checked for completeness and consistency before analysis. Descriptive statistics, such as frequencies and percentages, were computed for categorical variables, and means with standard deviations for continuous variables.

The chi‐square (*χ* ^2^) test was applied to assess associations between categorical variables. A *p* value of <0.05 was considered statistically significant.

## 3 Results

### 3.1 Socioeconomic Characteristics of Respondents (Table 1)

**FIGURE 2:**
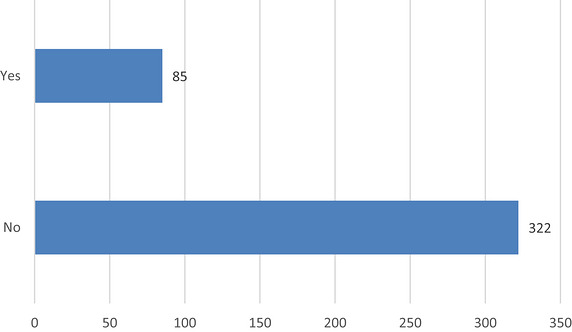
Exclusive breastfeeding status of children.

A total of 407 participants were included in the study, yielding a response rate of 96.0%. The distribution of respondents was relatively balanced across the IDP camps, with Kanyaruchinya (22.4%) and Bulengo (21.4%) contributing the highest proportions, whereas Don Bosco had the lowest representation (15.2%). Most children were aged 13–24 months (50.4%), followed by those aged 25–36 months (30.2%), with slightly more females (52.1%) than males (47.9%). The majority of children were born at term (57.5%), although over a quarter of respondents (27.5%) were uncertain about prematurity status. More than half of households had fewer than five members (54.1%), and nearly all had fewer than five children under five (99.0%). The respondents were predominantly young adults aged 20–29 years (57.2%), followed by those aged 30–39 years (23.8%), and educational attainment was generally low, with most having primary education (55.5%) or no formal education (28.3%), and only a small proportion attaining secondary (14.5%) or tertiary education (1.0%). In terms of marital status, married women constituted the largest group (37.3%), followed by single (30.0%) and widowed (24.1%) respondents, whereas the majority of children (83.5%) did not live with both parents. Economically, most households relied on trade (78.9%) as their main source of income, with fewer depending on agriculture (11.8%) or humanitarian assistance (7.4%), reflecting a predominantly informal and economically vulnerable population. It can be seen from the data of the survey that most of the children, 79.1% (*n* = 322), were not exclusively breastfed for 6 months, and only 20.9% (*n* = 85) were (as shown in Figure 2).

**FIGURE 3:**
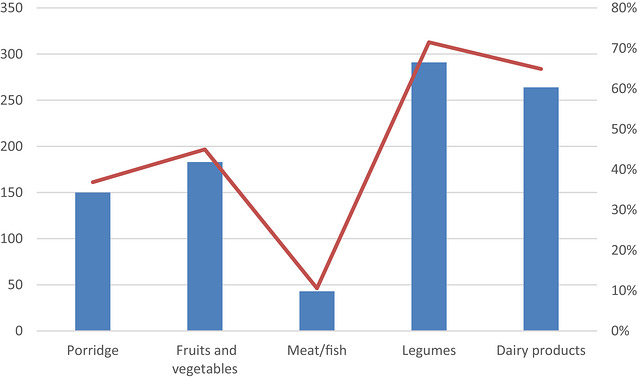
Types of foods/complementary food given to children.

Figure 3 on the types of foods regularly given to children indicates varied dietary practices among the population. The most provided food group is legumes, consumed by 71% (*n* = 291) of the children, followed closely by dairy products at 65% (*n* = 264). Fruits and vegetables are also common in children's diets, with 45% (*n* = 183) of respondents indicating regular provision. Porridge is given to 37% (*n* = 150) of the children, suggesting it is a staple for some households. In contrast, meat or fish is the least frequently given, reported by only 11% (*n* = 43) of carers.

### 3.2 Determinants of EBF (Table 2)

Many significant associations between EBF and different sociodemographic and household characteristics were found. EBF was strongly connected to the child's age, with the most current findings for EBF in children from 49 to 60 months old (71.4%) and the least in those from 25 to 36 months old (18.7%). Age had a significant connection (*χ* ^2^ = 18.8) with the responses, as the youngest mothers and those who were 20–29 years old breastfed their babies more frequently than the older mothers. Respondents with university degrees had the highest rate of EBF (100%); those with secondary education managed to do so in about a third of cases (33.9%), whereas educational status below the secondary level resulted in even lower rates. The chance of EBF was increased for married women and decreased for divorcees: 84.9% of married women practiced EBF, but only 54.3% of divorcees did. Moreover, the number of parents in the child's family was associated with EBF, as children with two parents were more likely to be breastfed (29.9%) than those without both parents (19.1%).

**TABLE 1:** Socioeconomic characteristics of respondents (407).

Variable	Category	Frequency (*N*)	Percentage
Name of the IDP camp	Rusayo	82	20.1
	Bulengo	87	21.4
	Don Bosco	62	15.2
	Kanyaruchinya	91	22.4
	Lushagala	85	20.9
Child's age	0–12 months	58	14.3
	37–48 months	14	3.4
	13–24 months	205	50.4
	25–36 months	123	30.2
	49–60 months	7	1.7
Gender of the child	Female	212	52.1
	Male	195	47.9
Prematurity status of the child at birth	Don't know	112	27.5
	No	234	57.5
	Yes	61	15.0
Number of people in the household	Less than 5	220	54.1
	More than 5	187	45.9
Children under five in household	Less than 5	403	99.0
	More than 5	4	1.0
Age of the respondent	Under 20 years	58	14.3
	20–29 years	233	57.2
	30–39 years	97	23.8
	40–49 years	18	4.4
	50 years and over	1	0.2
Level of education	No education	115	28.3
	Primary	226	55.5
	Primary (duplicate entry)	3	0.7
	Secondary	59	14.5
	University	4	1.0
Marital status of mother	Divorcee	35	8.6
	Bachelor	122	30.0
	Bride	152	37.3
	Widow	98	24.1
Child lives with both parents	No	340	83.5
	Yes	67	16.5
Main source of family income	Agriculture	48	11.8
	Humanitarian aid	30	7.4
	Humanitarian aid (duplicate)	1	0.2
	Other (specify)	7	1.7
	Trade	321	78.9

Abbreviation: IDP, internally displaced persons.

**TABLE 2:** Chi‐square test of association of determinants of exclusive breastfeeding for the first 6 months among children under five in internally displaced persons camps.

		Exclusive breastfeeding status			
Variable	Categories	No (*N*, %)	Yes (*N*, %)	*χ* ^2^	*p* value
IDP camp	Rusayo Bulengo Don Bosco Kanyaruchinya Lushagala	65 (79.3) 70 (80.5) 48 (77.4) 70 (76.9) 69 (81.2)	17 (20.7) 17 (19.5) 14 (22.6) 21 (23.1) 16 (18.8)	0.688	0.953
Child's age	0–12 months 37–48 months 13–24 months 25–36 months 49–60 months	46 (79.3) 8 (57.1) 166 (81.0) 100 (81.3) 2 (28.6)	12 (20.7) 6 (42.9) 39 (19.0) 23 (18.7) 5 (71.4)	15.7	0.003
Child's gender	Female Male	166 (78.3) 156 (80.0)	46 (21.7) 39 (20.0)	0.177	0.674
Premature birth	Don't know No Yes	95 (84.8) 174 (74.4) 53 (86.9)	17 (15.2) 60 (25.6) 8 (13.1)	7.64	0.022
People in household	5 >5	182 (82.7) 140 (74.9)	38 (17.3) 47 (25.1)	3.78	0.052
Children under five	<5 >5	319 (79.2) 3 (75.0)	84 (20.8) 1 (25.0)	0.0414	0.839
Respondent's age	<20 20–29 30–39 40–49 ≥50	50 (86.2) 194 (83.3) 68 (70.1) 10 (55.6) 0 (0.0)	8 (13.8) 39 (16.7) 29 (29.9) 8 (44.4) 1 (100)	18.8	<0.001
Education level	No education Primary Secondary University	94 (81.7) 189 (83.6) 39 (66.1) 0 (0.0)	21 (18.3) 40 (16.4) 20 (33.9) 4 (100)	23.8	<0.001
Marital status	Bachelor Bride Divorcee Widow	91 (74.6) 129 (84.9) 19 (54.3) 83 (84.7)	31 (25.4) 23 (15.1) 16 (45.7) 15 (15.3)	19.5	<0.001
Child lives with both parents	No Yes	275 (80.9) 47 (70.1)	65 (19.1) 20 (29.9)	3.90	0.048
Main income source	Agriculture Humanitarian aid Trade Other	32 (66.7) 15 (48.4) 269 (83.8) 6 (85.7)	16 (33.3) 16 (51.6) 52 (16.2) 1 (14.3)	28.3	<0.001

Abbreviation: IDP, internally displaced persons.

Other factors that showed significant associations include premature birth status (*χ* ^2^ = 7.64 and *p* = 0.022), where children not born prematurely had higher EBF rates. The family's main source of income was strongly linked to EBF practices (*χ* ^2^ = 28.3 and *p* < 0.001), with the highest EBF rates reported among family's dependent on humanitarian aid (51.6%) and the lowest among those relying on trade (16.2%). On the other hand, some variables did not show statistically significant associations with EBF. These include the specific IDPs camp of residence (*χ* ^2^ = 0.688 and *p* = 0.953), the gender of the child (*χ* ^2^ = 0.177 and *p* = 0.674), the number of children under five in the household (*χ* ^2^ = 0.0414 and *p* = 0.839), and the number of household members (*χ* ^2^ = 3.78 and *p* = 0.052), although the latter was close to the significance threshold.

## 4 Discussion

### 4.1 Key Findings

Of the children living in IDPs camps in Eastern DRC, only 20.9% were exclusively breastfed for the first 6 months, says a survey. There was a strong link between the mother's age, education, and family status, whether the child was premature, if the parents lived together, and the way the family earned their living on the practice of EBF. A higher number of younger mothers below 30, women with secondary or tertiary education, and women who were married were seen to exclusively breastfeed their babies. Those kids, who were more likely to be exclusively breastfed, were born at term and lived together with both parents. Outstanding EBF results were shown by people receiving humanitarian assistance rather than by people relying on commerce. It was found that EBF was scarcely linked to the camp attended by the child, the child's gender, the size of the home, or the general number of children 5 years old or younger in the home. This shows why it is important to create special programs and teach mothers from vulnerable and displaced groups to help improve nursing care. This is likely to assist mothers in comprehending the benefits of nursing and empower them to make informed judgments.

### 4.2 Interpretation

The EBF rate of this study, 20.9%, is well below national and international standards. The 2021 D. R. Congo Demographic and Health Survey (DHS) indicates that the nationwide EBF rate for children under 6 months is approximately 48%, implying that children in IDPs settings have greater challenges to optimal nursing practices [7, 9]. The WHO recommends a global EBF rate of at least 50%, with a target of 70% by 2030. The highlighted disparity underscores the susceptibility of displaced individuals [10].

Concerning childbearing age, our findings align with studies from Ethiopia and Nigeria, indicating that younger mothers are more inclined than their older counterparts to adhere to EBF practices [11, 12]. This may indicate a heightened involvement of younger women in maternal health efforts or an enhanced responsiveness to health education. Women with higher education had much superior adherence to EBF [13]. This aligns with studies by Koray et al., which indicate that maternal education is a strong predictor of EBF in sub‐Saharan Africa.

EBF was also affected by marital status; for example, married women exhibited the highest adherence rates [14, 15]. A study conducted in South Sudan indicates that persistent EBF is positively connected with spousal support [16]. The presence of both parents may provide social and emotional stability, enabling mothers to maintain their nursing practices.

EBF had a negative correlation with premature delivery, as evidenced by a study conducted in Uganda, which indicated that mothers of preterm infants were less inclined to initiate or sustain EBF due to perceived feeding difficulties. The family's primary income source further influenced EBF [17, 18]. Interestingly, families receiving humanitarian relief reported superior EBF practices compared to those reliant on trade, possibly due to targeted nutritional interventions by NGOs [19, 20]. Mothers employed in commerce may face conflicting obligations or restricted time that hinders breastfeeding.

Factors such as the specific IDPs camp, the child's gender, and the number of young children had no significant link with EBF, corroborating findings from conflict settings in Syria, where structural limitations typically supersede demographic variations [21]. Our findings indicate that nursing practices among displaced individuals are influenced by the intersection of family dynamics, economic instability, and educational attainment. Standard public health initiatives may need to be modified to address the specific challenges faced by IDPs.

### 4.3 Strengths and Implications of the Study

The study gives an in‐depth analysis of the factors that influence EBF among the population of IDPs. Having more than 300 people from five intensive care camps participate, we were able to present a balanced view of nursing practices for high‐risk people. It points out that some flexible characteristics, for example, mother's education, being married, and income status, help develop effective health guidance efforts. If maternal education and nursing education are promoted in emergencies, it can boost the number of people practicing EBF. It is noted in the study that we should include and encourage young mothers by providing community‐based support. As being surrounded by both parents and EBF are linked, male partners should also be involved in intervention programs. There are effects of policy development as well as how services are delivered. The findings support the right allocation of resources and shape nutrition programs for IDPs camps. Additionally, authorities in health care may choose to involve displaced persons more in their programs for maternal and child health and nursing promotion. The results support the need to improve assistance for mothers who want to breastfeed in emergency and resettlement places.

### 4.4 Limitations of the Study

Despite giving us important new information, this study still has some limitations. As the cross‐sectional approach is used, no direct connections between sociodemographic variables and EBF can be established. Moreover, the information people give on their nursing practices might be affected by wanting to look good or forgetting certain things, especially about EBF. Additionally, because of security and difficulty in accessing information, we could not study maternal mental health or healthcare services that might affect nursing mothers. We should have also questioned mothers about their attitudes and information on nursing, because this would provide more insight into their actions. There could have been possibility of recall bias in the case of collecting data about giving birth in the last 5 years and 6 months of EBF. Even though the study involved different perspectives, the results could be limited for IDPs in places outside the region examined. There could have been some potential selection bias due to the exclusion of newer camp residents or other humanitarian settings. However, the study sheds light on how often EBF is given to a group that has not been studied much.

### 4.5 Recommendations

Our findings prompt us to recommend many targeted strategies to enhance EBF rates in IDP settings. As a first step, making mothers understand the value of EBF helped the programs improve educational and health measures in the camps. In addition, humanitarian groups should include support for nursing when they give nutrition and health services, as well as involve peer counseling, mother‐to‐mother groups, and training for community health workers. Third, care for the mothers of preterm babies ought to consist of specialized programs for breastfeeding and meeting their particular needs. Support from spouses at home would improve the nursing experience, especially when family members, particularly males, get involved. Luckily, offering food aid or support for earning an income will assist mothers who are already practicing EBF. Lastly, individuals in charge and aid organizations can help set up IDPs camps with areas for breastfeeding and easy access to maternal health services.

### 4.6 Perspectives

This study looks at how nursing works in countries affected by situations of war and crises. It is needed to realize how such settings affect the behaviors of mothers and children, as there are currently more than 114 million people around the world who have been forcibly displaced. We saw that people who relocate experience greater hardships and tend to stop EBF. Addressing this issue necessitates context‐specific, equity‐focused activities that consider the lived experiences of displaced women, rather than relying just on conventional public health messages. Incorporating maternal and child health into humanitarian response plans is now essential rather than discretionary. Further longitudinal and qualitative research is urgently required to examine the impact of social, cultural, and psychological factors during displacement on newborn feeding choices. Facilitating multi‐sectoral collaborations among international organizations such as WHO, UNICEF, and UNHCR will ensure that breastfeeding support is prioritized in emergency nutrition plans [22]. Future programming must prioritize not just survival but also long‐term developmental outcomes for children in crisis contexts. This aids in upholding global health standards as well as the dignity and rights of displaced women and children [23, 24, 25]. It is also paramount to consider displaced populations where health infrastructure is limited and emphasize the role of counseling during antenatal/postnatal care. A case–control study done in Ethiopia suggests areas where intervention could improve EBF rates and offers a solid foundation of evidence for arguing that targeted maternal support is essential even in fragile, displaced settings. This can be achieved by enhanced health guidance, training for healthcare workers, and psychosocial support [26].

## 5 Conclusion

Eastern DRC's IDPs camps in Goma have very few mothers who are exclusively breastfeeding their children, which is less than half of what is considered the standard by both national and international guidelines. We found that the use of EBF is mostly shaped by factors such as a mother's age, level of education, and marital status, if the child was born prematurely, whether parents live together, and the main family income source. These determinants show that context‐specific strategies should aim to boost education for mothers, get men more involved, and always involve breastfeeding when helping people affected by displacement. More women getting humanitarian support are breastfeeding their babies, so it seems that special efforts can help a lot during crises. All cases considered, this research underlines that prompt and harmonized effort is required to care for and encourage breastfeeding in people who have been displaced. In the future, helping children in humanitarian situations should be guided by an approach that includes several parts of society, is culturally aware, and respects equity. This approach will help to cut child illness and respect the basic rights of mothers and their kids. The findings should directly warrant humanitarian breastfeeding support strategies in IDPs settings and improve EBF.

## Author Contributions

Conceptualization: **Aymar Akilimali**, **Christian Tague**, and **Elie Kihanduka**. Data curation: **Alimasi Kashafali**, Elie Kihanduka, and Medical Research Circle (MedReC) Collaborators. Formal analysis: Frank Odonkor Offei and **Amidu Alhassan**. Funding acquisition: Aymar Akilimali, Alimasi Kashafali, Elie Kihanduka, and **Rodrigue Fikiri Bavurhe**. Investigation: Alimasi Kashafali and Aymar Akilimali. Methodology: Aymar Akilimali. Project administration: Aymar Akilimali and **Michée Sanza Kanda**. Resources: Elie Kihanduka and **Calvin R. Wei**. Supervision: Rodrigue Fikiri Bavurhe, **Freddy Zihindula**, and **Amos Kipkorir Langat**. Validation: Calvin R. Wei and **Francois Rhugendabanga**. Visualization: Elie Kihanduka and **Innocent Mufungizi**. Writing – original draft: **Hugues Cakirwa**, **Fabien Balagizi**, Aymar Akilimali, Innocent Mufungizi, **Abel Aizeque**, and **Kanza Farhan**. Writing – review and editing: All Authors. Final approval of manuscript: All Authors.

## Funding

The authors have nothing to report.

## Disclosure

All authors have read and approved the final version of the manuscript. Aymar Akilimali and Alimasi Kashafali have full access to all of the data in this study and take complete responsibility for the integrity of the data and the accuracy of the data analysis.

## Ethics Statement

The study protocol was reviewed and approved by the Ethics Review Board of the Medical Research Circle (MedReC) in D. R. Congo following the relevant approval letter, Ref/ERB/004/ 2025. The study design incorporated the separation of patient identification data by confidentiality codes to maintain anonymity and protect the privacy of the participants. This study was performed in accordance with the ethical standards as laid in the 1964 Declaration of Helsinki and its later amendments or comparable ethical standards.

## Consent

Informed consent was obtained from all participants involved in the study.

## Conflicts of Interest

The authors declare no conflicts of interest.

## Transparency Statement

Alimasi Kashafali affirms that this manuscript is an honest, accurate, and transparent account of the study being reported; that no important aspects of the study have been omitted; and that any discrepancies from the study as planned have been explained.

## Data Availability

The authors have nothing to report.
